# From Food to Humans: The Toxicological Effects of *Alternaria* Mycotoxins in the Liver and Colon

**DOI:** 10.3390/jox15060205

**Published:** 2025-12-02

**Authors:** Rita Sofia Vilela, Francisco Pina-Martins, Célia Ventura

**Affiliations:** 1 Department of Human Genetics, National Institute of Health Doutor Ricardo Jorge, 1649-016 Lisbon, Portugal; rita.vilela@insa.min-saude.pt; 2 Departamento de Biologia, Faculdade de Ciências, Universidade de Lisboa, Campo Grande, 1749-016 Lisboa, Portugal; 3 Centre for Ecology, Evolution and Environmental Changes (CE3C) & CHANGE–Global Change and Sustainability Institute, Faculdade de Ciências, Universidade de Lisboa, Campo Grande, 1749-016 Lisboa, Portugal; frmartins@ciencias.ulisboa.pt; 4 Departamento de Engenharia Química e Biológica, Escola Superior de Tecnologia do Barreiro, Instituto Politécnico de Setúbal, Rua Américo da Silva Marinho, 2839-001 Lavradio, Portugal; 5 Comprehensive Health Research Centre (CHRC), NOVA Medical School, Universidade NOVA de Lisboa, 1150-190 Lisbon, Portugal

**Keywords:** alternariol, alternariol monomethyl ether, tenuazonic acid, altertoxin-I, altenuene, tentoxin, cytotoxicity, genotoxicity

## Abstract

*Alternaria* mycotoxins represent a significant and emerging concern in the field of food safety due to their widespread occurrence in diverse food and feed commodities, including cereals, tomatoes, oilseeds, and dried fruits. Among these, alternariol (AOH), alternariol monomethyl ether (AME), tenuazonic acid (TeA), and altertoxin-I (ATX-I) are the most frequently detected, often co-occurring at varying concentrations, thereby increasing the complexity of exposure and risk assessment. The gastrointestinal tract (GIT) is a crucial target of these toxins, as well as the liver, particularly considering its detoxifying role. Nevertheless, despite being a source of possible gastrointestinal and hepatic toxicity, there is still scarce data on the toxicokinetics of *Alternaria* toxins, on their mode of action, and respective toxic effects. To date, in vitro studies have shown that different *Alternaria* mycotoxins exhibit diverse toxicological effects, which may be dependent on their chemical structure. AOH and ATX-I have shown genotoxicity and cytotoxicity, mainly through interaction with the DNA and apoptosis, respectively. Tentoxin (TEN) has displayed hepatotoxic potential via impairment of detoxification pathways, and altenuene (ALT) has revealed lower toxicity. In vivo, AME and ATX-II revealed genotoxicity, while AOH and ATX-I showed context-dependent variability in their effects. Altogether, this review emphasizes that there is still a great lack of knowledge on these mycotoxins and an urgent need for more comprehensive toxicological and occurrence data to support proper risk assessment and, ultimately, regulatory decision-making.

## 1. Introduction

Molds can negatively impact agricultural products through discoloration, nutrient loss, reduced germination, and contamination with mycotoxins—secondary metabolites that are toxic to vertebrates and other animals, such as crustaceans [1,2,3,4,5,6]. These compounds are produced primarily by filamentous fungi of the genera *Alternaria*, *Aspergillus*, *Fusarium*, and *Penicillium* [7].

Environmental and anthropogenic factors, such as climate change, fungal interactions, and global food trade, further complicate mycotoxin contamination. Temperature, humidity, and insect activity can promote fungal growth and, thus, the presence of these secondary metabolites in food [3,4,7]. Intensified agricultural practices and food market globalization also contribute to the spread of toxigenic fungi into new regions [8]. In a large-scale global survey conducted between 2008 and 2017 across 100 countries, the analysis of over 74,000 raw material samples for food and feed revealed that 88% were contaminated with at least one mycotoxin, and 64% contained two or more [5]. Therefore, mycotoxins carry substantial economic costs, as contaminated crops and animal feed often require disposal, reducing food availability and affecting livestock productivity [6,8]. In animals, bioaccumulation of toxins can occur through contaminated feed, potentially introducing mycotoxins into the human food chain [8,9]. Moreover, animals, like humans, are frequently exposed to multiple mycotoxins simultaneously due to mixed feed ingredients, and unexpected combined effects may happen. Despite stricter food safety regulations in developing countries, mycotoxin contamination can still occur. Mycotoxins may remain in food products even after visible fungal growth is no longer present, resulting in hidden contamination in both raw commodities (e.g., fruits and vegetables) and processed products such as flour or animal feed [10]. These findings underscore the pervasive nature of mycotoxin contamination and the necessity for effective mitigation strategies.

Human exposure to mycotoxins occurs predominantly through the oral route by ingestion of contaminated food, though dermal and inhalation routes are also relevant, especially in occupational settings dealing with organic dust, e.g., animal farms or bakeries, and in moist indoor environments favoring fungal growth [7,11,12]. Mycotoxins have been associated with a range of toxic effects in humans, including nephrotoxicity, hepatotoxicity, immunotoxicity, carcinogenicity, and teratogenicity [4,5,6,7,10]. The severity of their health effect depends on the type of mycotoxin, exposure level, and duration, as well as individual factors such as age, health status, and diet [13].

Among the mycotoxin-producing fungi, *Alternaria* is emerging as a particular concern due to its frequent occurrence in a wide range of crops and its capacity to synthesize numerous mycotoxins. The *Alternaria* genus comprises approximately 280 fungal species, exhibiting diverse ecological roles such as saprophytes, endophytes, and, predominantly, phytopathogens [14]. These fungi infect a wide range of substrates, including crops of high agricultural importance such as cereals and oilseeds, as well as vegetables (e.g., broccoli, carrots, potatoes) and fruits (e.g., tomatoes, citrus, and apples) [14,15]. Similarly to other fungi, many *Alternaria* species act as plant pathogens, causing diseases such as leaf spots and stem infections, which primarily occur during the pre-harvest phase and can significantly reduce crop yield [15]. During post-harvest storage, these fungi may continue to proliferate, leading to the accumulation of mycotoxins in the edible parts of the plants [15,16]. Additionally, *Alternaria* species are capable of growing at low temperatures, maintaining their toxigenic potential even in refrigerated storage conditions [16].

**Figure 1:**
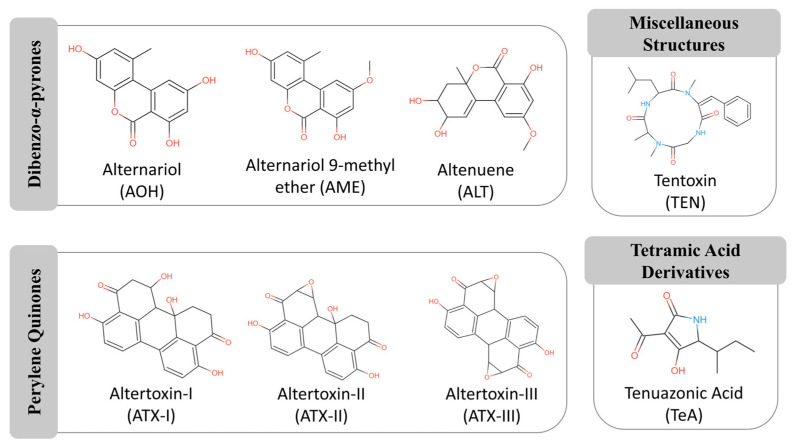
Chemical structures of representative *Alternaria* mycotoxins, grouped according to their structural classes. The main groups include dibenzo-α-pyrones (e.g., alternariol, alternariol monomethyl ether), perylene quinones (e.g., altertoxin I, II), tetramic acid derivatives (e.g., tenuazonic acid), and other miscellaneous compounds. The functional groups responsible for biological activity are highlighted in red (e.g., hydroxyl, ketone) and blue (nitrogen). Adapted from MycoCentral database [17].

Over 70 secondary metabolites have been identified in this genus, although only a subset has demonstrated toxicological relevance in humans and animals [9,13]. They are often heat-stable and resistant to conventional food processing, making their detection and mitigation particularly challenging for food safety authorities [4,6,7,15]. In this regard, the European Food Safety Authority (EFSA) has identified alternariol (AOH), alternariol monomethyl ether (AME), tenuazonic acid (TeA), altenuene (ALT), and tentoxin (TEN) as the most relevant compounds [13]. Among these, TeA and dibenzopyrone derivatives (AOH, AME, ALT) are the most prevalent food contaminants, together with altertoxins (ATXs) [13,15]. The chemical structures of these mycotoxins are grouped by class in Figure 1.

Despite their widespread occurrence in food and feed and toxicological potential, knowledge on human exposure to *Alternaria* mycotoxins and their toxicity is limited, impairing risk assessment to protect public health [15]. As such, there is growing interest in understanding the consequences of chronic dietary exposure [14].

In this review, we provide an updated overview of the occurrence, toxicokinetics, and intestinal and hepatic toxicological effects of major *Alternaria* mycotoxins—AOH, AME, TeA, TEN, ALT, and ATXs. Special emphasis is given to their genotoxic and cytotoxic effects, based on both in vitro and in vivo studies.

## 2. *Alternaria* Mycotoxins Occurrence

As already mentioned, *Alternaria* species can contaminate a wide range of agricultural commodities—such as grains, maize, tomatoes, sunflower seeds, and dried fruits—at multiple stages of the production chain, including pre-harvest, harvest, post-harvest handling, and storage. Suboptimal conditions during these stages, such as high humidity and poor ventilation, can promote fungal proliferation and increase the risk of mycotoxin production [9]. While carry-over of mycotoxins from feed to animal-derived foods is well established for several toxins groups—particularly aflatoxins and *Fusarium* toxins—the extent to which *Alternaria* mycotoxins may transfer through the food chain remains insufficiently characterized. Nonetheless, their presence in feed suggests that this possibility warrants further investigation [18,19,20]. Numerous surveys have reported not only the widespread occurrence of individual mycotoxins, but also their simultaneous presence within the same samples, emphasizing the importance of comprehensive monitoring and regulation.

Following EFSA’s 2011 designation of *Alternaria* mycotoxins as emerging food contaminants, European countries have reported numerous contamination cases, while a growing number of studies have documented similar findings in Asia—especially in China, which is among the world’s leading producers and traders of tomatoes and cereals [21]. Focusing on specific commodities, tomato products are among the food matrices most frequently contaminated with *Alternaria* mycotoxins. Large-scale monitoring revealed widespread presence of AOH, AME, and TeA in various tomato derivatives, with detection rates ranging from 20% to 100% and concentrations reaching up to 462 µg/kg [22]. In The Netherlands TeA was identified in all dried fig samples, 80% of sunflower seeds, and 60% of tomato products, with levels of up to 2345 µg/kg [23]. In samples from Belgium, TeA was present in over 70% of tomato products, along with frequent detection of AOH, AME, TEN, and ALT—particularly in concentrates, where processing may increase toxin levels [24]. In contrast, AOH and AME were not detected in Brazilian tomato samples, although TeA was found in some sauces and concentrates [25]. Marked regional differences are evident in a study where exceptionally high levels of AOH (up to 8800 µg/kg), AME (1700 µg/kg), and TeA (4000 µg/kg) in Argentine tomato purée were detected [26].

**Figure 2:**
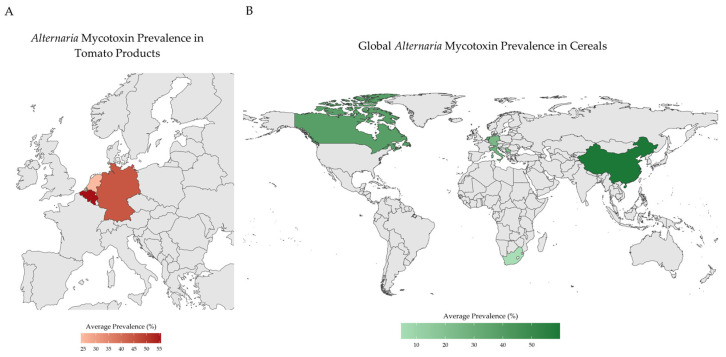
Summary of the reported *Alternaria* mycotoxins contamination trends. (**A**) *Alternaria* mycotoxin prevalence in tomato products in Europe; (**B**) *Alternaria* mycotoxin prevalence in cereals worldwide. Created in R 4.5.2 [31].

Cereal grains, especially wheat, also show consistent contamination. Two mycotoxins (AOH and AME) were found in 31% and 26% of durum wheat samples from Italy, with concentrations of up to 121 µg/kg [27]. In Serbia, TeA was reported as the most prevalent mycotoxin in wheat, present in 68.5% of samples and reaching 2676 µg/kg, while AOH and AME were less common [28]. Similarly, in Chinese samples, TeA was detected in all 370 wheat kernel samples, with concentrations up to 3330 µg/kg and co-occurrence of TEN, AOH, and AME in up to 95% of cases [29]. Although maize is generally less affected, *Alternaria* toxins are still detected. In Africa, AME and ALT were detected in 7.1% of maze samples and both AOH and AME were often below the quantification limits; ATX-I was measured in one sample at 43 µg/kg, indicating a lower, but still notable, presence [30]. A visual summary of reported *Alternaria* mycotoxin prevalence in tomato products (Figure 2A) and cereal grains (Figure 2B) is presented in Figure 2.

Comparative data indicate notable regional variability in *Alternaria* mycotoxin contamination, with higher concentrations frequently reported in Asian samples—particularly from China—compared to those from Europe [26]. The globalization of agricultural trade has intensified concerns about the transboundary movement of mycotoxins, particularly those produced by fungi of the genus *Alternaria*. Given the extensive domestic production and participation of China in international trade, monitoring of *Alternaria* toxins in Chinese commodities has increased to ensure compliance with importing countries’ food-safety requirements [13]. Altogether, the globalization of food trade underscores the potential for cross-border dissemination of *Alternaria* toxins and highlights the importance of coordinated global monitoring and regulatory alignment to reduce regional exposure risks.

The prevalence and concentration of these toxins appear to be influenced by climatic conditions, post-harvest storage, and processing practices. For instance, processing techniques for citrus-based products in China have been linked to higher toxin levels than those reported abroad [32]. The lack of harmonized international regulations further compounds these risks, allowing contaminated goods to circulate through global supply chains.

Processed foods have also been extensively investigated regarding *Alternaria* mycotoxins contamination. In Germany, out of 96 processed food samples—including tomato-based products, juices and baked goods—91.2% contained at least one mycotoxin [33]. AME (68%), TeA (67%), AOH (60%), and TEN (48%) were the most frequently found. Co-contamination was common: 26% of samples contained the four toxins simultaneously, especially in fruit juices and bakery items, which are major contributors to dietary exposure [33].

Moreover, there is growing evidence that modified forms of *Alternaria*-derived mycotoxins—specifically glycosylated (e.g., glucoside) and sulfated conjugates of compounds like AOH and AME—can occur in foodstuffs. These modified toxins are believed to arise from host plant detoxification mechanisms, as glycosylation-AOH-3-glucoside (AOH-3-G), AOH-9-glucoside (AOH-9-G), and AME-3-glucoside (AME-3-G)-or fungal/plant transformation by sulfation-AOH-3-sulfate (AOH-3-S) and AME-3-sulfate (AME-3-S)-rather than primary fungal toxin production [34]. For example, AOH-3-G, AOH-9-G, AOH-3-S and AME-3-S have been detected in tomato products, with AOH-9-G and AME-3-S detected in tomato sauce at levels comparable to their parent toxins [35]. Walravens et al. (2016) also analyzed different tomato products and found AOH-3-S and AME-3-S at values of up to 8.7 µg/kg and 9.9 µg/kg, respectively, and no glucoside forms [36]. Modified *Alternaria* mycotoxins were also reported in apple-by-products, in which only AME-3-S was present in the raw material, but AOH-3-S and AOH-3-G were detected along the process; AME-3-G were not detected at any stage [37]. These findings suggest that actual dietary exposure to AOH, AME and other toxins may be underestimated, although their highly relevance for food safety because they may revert to the parent toxin in the gastrointestinal tract (via hydrolysis). Hence, the occurrence of sulfated and glycosylated AOH and AME in food commodities should be studied more intensively, although analytical methods for detecting them are scarce [34]. More research has to be conducted in this area, as information about the occurrence of modified forms of *Alternaria*-derived mycotoxins, as well as toxicity data, is still lacking.

Co-occurrence of multiple *Alternaria* toxins—reported in 44% of positive samples in South Korea—further raises concern due to potential additive or synergistic effects [38]. Indirect exposure through animal-derived products is also possible, as contaminated feed can transfer mycotoxins to livestock and ultimately to consumers. For example, in one study of 83 feed samples, AOH and AME were detected in 80% and 82% of samples with maximum levels of 221 µg/kg and 733 µg/kg feed, respectively [26]. TeA and TEN were also common, reaching 1983 and 76 µg/kg [26].

These findings underline the widespread occurrence of *Alternaria* mycotoxins across diverse food matrices, including tomato products, grains, sunflower seed, and processed foods, with marked variability influenced by geographic origin and processing methods. TeA frequently emerges as the dominant toxin, often co-occurring with AOH, AME, and TEN, particularly in processed tomato products, bakery goods, and fruit juices (Table 1).

As indicative levels have only been set for the foodstuffs for which sufficient occurrence data are available, the European Commission (EU 2022/553) recommended systematic monitoring of AOH, AME, and TeA in key food matrices—such as tomato products, seeds and cereal-based products—and reporting results to EFSA [39]. This initiative provides a framework for assessing exposure, identifying contributing factors, and guiding food safety measures [39].

**Table 1:** Summary of prevalence and concentration levels of *Alternaria* mycotoxins reported in food samples. Data were compiled from published occurrence studies covering various food matrices. Prevalence is expressed as the percentage of positive samples relative to the total number analyzed in each study. Concentration (µg/kg food) refers only to quantified values.

	Country	Detection Method	N	Prevalence (%)	Concentration (µg/kg)		Ref
					Mean	Max	
Cereals							
ALT	Germany	HPLC-MS/MS	9	0	-	-	[33]
		HPLC	267	2.6	-	197	[40]
	South Africa	HPLC-MS/MS	42	7.1	8.7	13	[30]
AOH	Canada	HPLC-MS/MS	44	9	-	8	[41]
	China	LC-ESI-MS/MS	370	47	12.9	74.4	[29]
	Germany	HPLC-MS/MS	9	89	-	<0.6	[33]
		HPLC	1064	8.1	77	832	[40]
	Italy	LC-MS/MS	74	31	11	121	[27]
	The Netherlands	LC-MS/MS	14	7.1	-	5.2	[42]
	Serbia	LC-MS/MS	92	12	18.6	48.9	[28]
		QuEChERS LC-MS/MS	20	20	3.3	5.3	[43]
	Slovenia	LC-MS/MS	433	11	155	1836	[44]
	South Africa	Micro HPLC-MS/MS	42	2.4	-	<0.005	[30]
AME	Canada	HPLC-MS/MS ESI-MRM	44	16	-	17	[41]
	China	LC-ESI-MS/MS	370	15	9.1	54.8	[29]
	Germany	HPLC-MS/MS	9	57	3.2	3.2	[33]
		HPLC	1064	3.1	77	905	[40]
	Italy	LC-MS/MS	74	26	7	48	[27]
	The Netherlands	LC-MS/MS	14	7.1	-	3.0	[42]
	Serbia	LC-MS/MS	92	6.5	39	70.2	[28]
		QuEChERS LC-MS/MS	20	10	2.2	2.3	[43]
	Slovenia	LC-MS/MS	433	6	148	1121	[44]
	South Africa	Micro HPLC-MS/MS	42	7.1	-	<0.005	[30]
ATX-I	Germany	HPLC-MS/MS	9	0	-	-	[33]
	South Africa	Micro HPLC-MS/MS	42	2.4	43	43	[30]
TeA	China	LC-ESI-MS/MS	370	100	289	3330.7	[29]
	Germany	HPLC-MS/MS	9	100	140	210	[33]
		HPLC	1064	30.2	-	4224	[40]
	Serbia	LC-MS/MS	92	68.5	92.4	2676	[28]
	Slovenia	LC-MS/MS	433	26	170	2277	[44]
TEN	Canada	HPLC-MS/MS ESI-MRM	44	89	-	63	[41]
	China	LC-ESI-MS/MS	370	77	43.8	258.6	[29]
	Germany	HPLC-MS/MS	9	100	11	12	[33]
	The Netherlands	LC-MS/MS	14	100	6.0	14	[42]
	Serbia	QuEChERS LC-MS/MS	20	0	-	-	[43]
	Slovenia	LC-MS/MS	433	8	27	116	[44]
Tomato Products							
ALT	Belgium	UPLC-MS/MS	83	46	8.27	62	[36]
	Germany	HPLC-MS/MS	34	0	-	-	[33]
	The Netherlands	LC-MS/MS	8	0	-	-	[42]
		LC-MS/MS	43	0	-	-	[45]
AOH	Belgium	UPLC-MS/MS	83	81	4.13	41.6	[36]
	Germany	HPLC-MS/MS	34	71	13	25	[33]
	The Netherlands	LC-MS/MS	8	50	16	25	[42]
		LC-MS/MS	43	37	4.8	26	[45]
AME	Belgium	UPLC-MS/MS	83	66	1.47	6.1	[36]
	Germany	HPLC-MS/MS	34	79	2.5	7.4	[33]
	The Netherlands	LC-MS/MS	8	50	3.8	7.8	[42]
		LC-MS/MS	43	9	1.2	5.6	[45]
ATX-I	Belgium	UPLC-MS/MS	83	0	-	-	[36]
	Germany	HPLC-MS/MS	34	0	-	-	[33]
TeA	Belgium	UPLC-MS/MS	83	100	62.5	333.1	[36]
	Germany	HPLC-MS/MS	34	91	200	460	[33]
	The Netherlands	LC-MS/MS	8	100	202	462	[42]
		LC-MS/MS	43	60	63	344	[45]
TEN	Belgium	UPLC-MS/MS	83	41	1.17	8.9	[36]
	Germany	HPLC-MS/MS	34	26.5	-	<0.5	[33]
	The Netherlands	LC-MS/MS	8	0	-	-	[42]
		LC-MS/MS	43	0	-	-	[45]
Dried Fruits							
ALT	The Netherlands	LC-MS/MS	14	0	-	-	[45]
		LC-MS/MS	5	0	-	-	[42]
AOH	China	UPLC-MS/MS	220	2.3	12	27.4	[46]
	The Netherlands	LC-MS/MS	14	7	2.5	8.7	[45]
		LC-MS/MS	5	0	-	-	[42]
AME	China	UPLC-MS/MS	220	8.2	3	15	[46]
	The Netherlands	LC-MS/MS	14	0	-	-	[45]
		LC-MS/MS	5	0	-	-	[42]
TeA	China	UPLC-MS/MS	220	42.7	456.5	5665.3	[46]
	The Netherlands	LC-MS/MS	14	100	473	1728	[45]
		LC-MS/MS	5	100	1043	2345	[42]
TEN	China	UPLC-MS/MS	220	20.5	120.5	1032.6	[46]
	The Netherlands	LC-MS/MS	14	0	-	-	[45]
		LC-MS/MS	5	0	-	-	[42]
Sunflower products							
ALT	Germany	HPLC-MS/MS	11	9.1	-	<2.5	[33]
	The Netherlands	LC-MS/MS	21	0	-	-	[45]
		LC-MS/MS	5	0	-	-	[42]
AOH	Germany	HPLC-MS/MS	11	54.5	27	39	[33]
	The Netherlands	LC-MS/MS	21	5	5.4	36	[45]
		LC-MS/MS	5	0	-	-	[42]
AME	Germany	HPLC-MS/MS	11	63.6	11	21	[33]
	The Netherlands	LC-MS/MS	21	10	1.8	17	[45]
		LC-MS/MS	5	0	-	-	[42]
ATX-I	Germany	HPLC-MS/MS	11	9.1	-	<6.9	[33]
TeA	Germany	HPLC-MS/MS	11	100	420	490	[33]
	The Netherlands	LC-MS/MS	21	38	240	1350	[45]
		LC-MS/MS	5	100	223	449	[42]
TEN	Germany	HPLC-MS/MS	11	90.9	110	800	[33]
	The Netherlands	LC-MS/MS	21	0	-	-	[45]
		LC-MS/MS	5	20	-	5	[42]
Vegetable Oil							
ALT	Germany	HPLC-MS/MS	19	0	-	-	[33]
AOH	Germany	HPLC-MS/MS	19	47.4	6	6	[33]
AME	Germany	HPLC-MS/MS	19	84.2	9.9	14	[33]
ATX-I	Germany	HPLC-MS/MS	19	0	-	-	[33]
TeA	Germany	HPLC-MS/MS	19	21.1	15	15	[33]
TEN	Germany	HPLC-MS/MS	19	47.4	11	11	[33]
Wine							
AOH	Germany	HPLC-MS/MS	25	96	2.13	7.65	[47]
	The Netherlands	LC-MS/MS	5	20	-	11	[42]
AME	Germany	HPLC-MS/MS	25	52	1.19	1.45	[47]
TeA	Germany	HPLC-MS/MS	25	88	10.73	60	[47]
	The Netherlands	LC-MS/MS	5	60	25	46	[42]
TEN	Germany	HPLC-MS/MS	25	40	1.17	1.47	[47]
Fruit Juices							
ALT	Germany	HPLC-MS/MS	23	4.3	-	<2.5	[33]
AOH	Germany	HPLC-MS/MS	23	56.5	3.1	16	[33]
		HPLC-MS/MS	78	27	4.08	4.31	[47]
AME	Germany	HPLC-MS/MS	23	43.5	1.8	4.9	[33]
		HPLC-MS/MS	78	5	1.28	1.54	[47]
ATX-I	Germany	HPLC-MS/MS	23	0	-	-	[33]
TeA	Germany	HPLC-MS/MS	23	52.2	21	250	[33]
		HPLC-MS/MS	78	63	3.67	19.2	[47]
TEN	Germany	HPLC-MS/MS	23	47.8	1	1	[33]
		HPLC-MS/MS	78	27	5.44	10.27	[47]

HPLC: High-performance liquid chromatography; HPLC-MS/MS: HPLC coupled with tandem mass spectrometry; HPLC-MS/MS ESI-MRM: HPLC–MS/MS using electrospray ionization with multiple reaction monitoring; LC-MS/MS: Liquid chromatography–tandem mass spectrometry; LC-ESI-MS/MS: LC–MS/MS using electrospray ionization; Micro HPLC-MS/MS: Micro-flow HPLC coupled with MS/MS; QuEChERS LC-MS/MS: QuEChERS extraction followed by LC–MS/MS analysis; UPLC-MS/MS: Ultra-performance liquid chromatography coupled with MS/MS.

## 3. Preventive Measures for Contamination Control

The prevention of *Alternaria* mycotoxin contamination begins with integrated pre-harvest strategies aimed at minimizing fungal colonization and toxin biosynthesis in crops. The adoption of Good Agricultural Practices (GAP)—including appropriate fertilization, irrigation, and pest control—reduces plant stress and thus the likelihood of fungal infection [48,49]. Agronomic measures such as crop rotation, the use of resistant or fungicide-treated seed varieties, and strict field hygiene further limit the persistence of *Alternaria* inoculum in the soil [50,51]. Environmental management also plays a decisive role: excessive rainfall or irrigation close to harvest increases susceptibility to *Alternaria*-related black point disease, while regionally adapted water management and the selection of tolerant cultivars can substantially reduce infection rates [52,53]. Recent advances also highlight the value of climate and environmental monitoring systems that integrate temperature and humidity data with disease forecasting models to anticipate contamination risks under changing climatic conditions [54].

Post-harvest interventions are equally critical to prevent fungal proliferation and secondary toxin formation. Maintaining optimal storage conditions—low humidity, stable temperature, and proper aeration—remains the most effective measure to suppress fungal growth [48,52,55]. Mechanical sorting, dehulling, and milling can physically remove contaminated grain fractions, while chemical and biological detoxification approaches, including ozone or chitosan treatments and microbial degradation, have shown promise in reducing toxin residues [56]. Because *Alternaria* species can grow at low temperatures, refrigerated storage and transport should also include regular fungal monitoring and strict hygiene to prevent cross-contamination [54,57]. Emerging technologies such as cold atmospheric plasma—shown to degrade AOH and AME by up to 62.8%—and antifungal active packaging materials incorporating biopolymers or natural extracts offer additional, innovative layers of protection [58,59].

Although *Alternaria* mycotoxins are generally considered heat-stable, recent evidence shows that food processing can partially reduce their levels depending on the matrix and processing conditions. Some processes, such as fermentation or baking, can reduce mycotoxin contamination but do not eliminate it entirely. For example, extrusion processing of whole-grain red sorghum flour reduced AOH by 60.6–62.1%, AME by 71–80%, TeA by 3.1–12.1% and TEN by 43–57% [60]. Similarly, dough fermentation and baking had no effect on TeA and AME content in bread, but reduced AOH content by 34.8% compared to its content in dough after kneading [61]. Moreover, it was found that the clarification step is key to reduce *Alternaria* toxins in apple concentrates, as mycotoxin quantities remained similar in clear and cloudy processes until the clarification step, in which all mycotoxins underwent a significant reduction to non-quantifiable levels, except TeA in one of the clarified final products [37]. In brewing, TeA was the only toxin to migrate into the final beer, while AOH, AME, TEN, and ATX I were mainly found in the spent grains, and AOH-3-S and AME-3-S metabolites were found in some processing steps [62]. These findings highlight that processing may shift, rather than eliminate, contamination—contributing to “hidden” residues in intermediate or by-products that can re-enter the food chain. Conjugated or “masked” mycotoxins can escape routine detection, posing additional challenges for exposure and risk assessments. Further studies are required to identify these to-date unknown conjugates through advanced new technology [63]. Moreover, several biological, physical, and chemical methods have been developed for the prevention or detoxification of mycotoxins in beverages and other processed foods during the postharvest period, although some are not so efficient or restricted due to safety concerns, possible degradation in the nutritional value of the products, and cost [64]. Although the complete elimination of *Alternaria* mycotoxins remains difficult, the integration of these preventive and post-harvest control measures can markedly reduce contamination levels. A continuous combination of good agricultural and manufacturing practices, coupled with environmental monitoring and technological innovation, is essential to mitigate dietary exposure risks and safeguard food safety [54,64].

## 4. Human Exposure to *Alternaria* Mycotoxins

The pervasive presence of *Alternaria* mycotoxins raises concerns about chronic dietary exposure. Even without specific toxicity data, Threshold of Toxicological Concern (TTC)–the maximum level of exposure expected to pose negligible risks–were set for the *Alternaria* mycotoxins: 2.5 ng/kg body weight (bw)/day for AOH and AME, and 1500 ng/kg bw/day [13,65]. According to EFSA (2016), average daily intakes have been estimated at 0.7–1.3 ng/kg bw/day for TEN and 1–15 ng/kg bw/day for AOH, with toddlers reaching up to 3.8–71.6 ng/kg/day for AOH—substantially above the TTC [22]. Similarly, in China, mean exposures reached 24.0 ng/kg bw/day for AOH and 6.09 ng/kg bw/day for AME, with high consumers and children exceeding the TTC limits; TeA exposure in children also slightly surpassed its TTC [66].

While average exposures in general population are typically near thresholds, infants, children, and high consumers of cereal-tomato-based foods remain the most at-risk groups, warranting continued monitoring and biomarker-based exposure studies. Infants and young children are the most vulnerable groups due to their higher food intake per body weight and immature detoxification systems [67,68]. In several infant foods (cereals and purees), estimated intakes for AOH and AME surpassed TTC values multiple times [69,70].

Moreover, TeA has been detected in human urine, confirming real-world exposure [25,71], and modified metabolites such as AOH-3-sulfate and AME-3-sulfate may contribute to underestimated total intake [35].

Currently, to our knowledge, no human biomonitoring studies have quantified modified forms of *Alternaria* mycotoxins—specifically glycosylated or sulfated conjugates such as AOH-9-G, AME-3-S, or related metabolites—in human biological fluids. Data on human exposure to glycosylated and sulfated *Alternaria* toxins remain absent, largely due to the lack of analytical standards and validated detection methods [34].

## 5. Toxicokinetics Data on *Alternaria* Mycotoxins

The toxicokinetics of *Alternaria* mycotoxins are still not fully understood, but available in vitro and in vivo studies provide valuable insights into their absorption, biotransformation, and systemic availability.

Regarding AOH, in vitro studies have shown both phase I hydroxylation (e.g., 2-, 4-, 8-, and 10-OH-AOH commonly formed) and phase II conjugation and glucuronidated and sulfated conjugates in human hepatic (HepG2, HepaRG) and intestinal (Caco-2) cells, as well as in liver and intestinal microsomes from different species [72,73,74]. Moreover, Caco-2 monolayers demonstrated moderate transepithelial transport, indicating that intestinal absorption of AOH is possible, although likely limited [73]. In vivo data consistently indicated low systemic bioavailability of AOH. Following oral administration of radiolabeled AOH (200–1000 mg/kg bw) in mice, approximately 90% of the dose was excreted in feces, 9% in urine, and only trace levels (0.006%) were detected in plasma, suggesting limited systemic circulation [72]. Similarly, in rats exposed to mixed *Alternaria* cultures, AOH was not detectable in plasma, and only small quantities were excreted in urine, primarily as AOH-3-O-sulfate [75]. These results indicate that while AOH is metabolically active, systemic exposure to the parent compound seems to be low, and fecal elimination predominates. Recent toxicokinetic data in pigs confirms this pattern: AOH exhibits low oral bioavailability (15%), reflecting limited absorption and extensive first-pass glucuronidation and sulfation. Hydroxylated catechol metabolites were also detected, suggesting potential toxicological relevance of these products [76].

Like AOH, AME undergoes extensive phase I hydroxylation (2-, 4-, 8-, and 10-OH-AME) and phase II glucuronidation and sulfation in hepatic and intestinal microsomal models [73,77,78,79,80]. Transport studies suggest limited permeability across intestinal barriers, consistent with low expected systemic availability. In rodent models, one study demonstrated that most of the orally administrated radiolabeled AME was excreted unchanged in feces, while urinary excretion accounted for <10% [81]. In another study, AME was also not detectable in plasma after exposure to mixed *Alternaria* extracts, and only small quantities of AME and AME-3-O-sulfate were recovered in urine (<3% of the dose) [82]. Accordingly, oral absorption appears poor and systemic exposure minimal, with fecal elimination dominating. Complementary pig toxicokinetic studies show that AME has very low oral bioavailability (9%) and undergoes rapid glucuronidation and sulfation, further supporting minimal systemic exposure to the parent toxin [76]. In rats exposed to a complex *Alternaria* extract, AME-3-sulfate—a metabolite not present in the administered mixture—represented up to 75% of urinary AME, confirming extensive phase II metabolism and highlighting its relevance as a potential biomarker [75].

In Caco-2 monolayers, ATX-I showed measurable transepithelial transport (~6% recovery in the basolateral compartment), suggesting potential for systemic absorption [83,84]. ATX-II displayed very low permeability, but was rapidly converted to ATX-I in both Caco-2 and HepG2 cells, indicating epoxide reduction as a major detoxification pathway. Due to its epoxide moiety, ATX-II is highly reactive, forming conjugates with thiols such as glutathione [85]. In rats orally administered with mixed *Alternaria* extracts, ATX-I was detected in plasma, urine, and feces at low levels (<4% of the dose), while ATX-II was not detected [75,82]. Exposure to isolated ATX-II resulted in systemic detection of ATX-I, confirming reductive de-epoxidation in vivo. Therefore, ATX-II likely acts locally in the gastrointestinal tract (GIT), while ATX-I represents the systemically relevant form. Recent in vivo studies also identified additional metabolites—including altersetin and altercrasin A—formed in the liver following exposure to toxin mixtures, suggesting previously unrecognized metabolic pathways [86]. These compounds may serve as biomarkers of exposure and indicate broader hepatic biotransformation capacity.

Data on ALT and TEN is very limited. In vitro data suggested that ALT is preferentially metabolized to 8-OH-ALT, which is subsequently glucuronidated [79,80]. In rat liver slices, approximately 50% of the administered ALT was present as glucuronides after 24 h, while ~10% underwent oxidation [87]. Moreover, ALT was not detected in plasma or urine, and only ~7% of the parent compound was recovered in feces following oral administration [75]. Thus, systemic exposure appears minimal, and extensive metabolic conversion may occur before elimination.

Studies on TEN are lacking. In vitro, rat liver microsomes show that TEN undergoes rapid hydroxylation and N-demethylation, producing multiple metabolites [88]. In rats exposed to mixed *Alternaria* extracts, TEN was largely excreted unchanged (~45% in feces within 24 h) and was not detectable in plasma, with <1% excreted in urine [75]. This suggests low oral bioavailability and efficient metabolic clearance.

In contrast to AOH and AME, however, pig toxicokinetic data indicate that TeA shows nearly complete oral bioavailability, underscoring substantial differences in systemic exposure among *Alternaria* toxins [76]. Additionally, mixture-exposure experiments in rats revealed substantial alterations in hepatic metabolism, including accumulation of short-chain acylcarnitines and downregulation of riboflavin, suggesting impaired β-oxidation and coenzyme-dependent pathways [86]. These effects were more pronounced after exposure to toxin mixtures than to isolated ATX-II, emphasizing the need to consider co-exposure in risk assessment. The same study also reported that several perylene quinones (e.g., ATX-II, STTX-III) were not detectable after administration, likely due to rapid degradation or transformation, whereas ATX-I and altenusin were found in excreta at low levels [75]. Enzymatic deconjugation assays further confirmed the presence of conjugated metabolites of AOH, AME, ATX-I, and ALP, highlighting the extensive role of phase II metabolism [75].

These findings suggest that across multiple *Alternaria* mycotoxins, oral bioavailability of parent compounds is generally low, with extensive metabolism and dominant fecal excretion. However, metabolic derivates—including hydroxylated and conjugated forms–may contribute to systemic toxicity, particularly for compounds such as ATX-II, which is locally reactive but converted to the more systemically persistent ATX-I (Table 2).

The identification of novel metabolites (e.g., altersetin, altercrasin A) and toxin-induced metabolic disturbances further underscores the importance of integrating metabolite profiles and mixture effects into toxicokinetic assessment frameworks [86].

With this knowledge about the absorption, distribution, metabolism, and excretion of *Alternaria* mycotoxins, the next section will examine the toxicity studies conducted on either the parent compounds or their metabolites, focusing on their cytotoxicity and genotoxicity.

**Table 2:** Overview of the toxicokinetic fate (absorption, metabolism, and excretion) of key *Alternaria* mycotoxins based on available in vitro and in vivo studies.

Mycotoxin	Absorption	Metabolism		Excretion	Ref.
		Phase I	Phase II		
AOH	Low systemic absorption	Hydroxylation	Glucuronidation and sulfation	Mainly fecal; minor urinary elimination	[72,73,74,75]
AME	Poor absorption	Hydroxylation	Glucuronidation and sulfation	Predominantly Fecal	[73,75,77,78,79,80,81,82]
ATX-I	Moderate permeability; detectable in plasma	Not reported	Not reported	Low urinary and fecal recovery	[75,82,83,84]
ATX-II	Poor permeability; not detectable in plasma	Reductive de-epoxidation to ATX-I	Glutathione conjugation	Not detected in urine or feces	[82,83,84,85]
ALT	Not detectable in plasma; limited absorption	Hydroxylation	Glucuronidation	Low fecal recovery	[75,79,87]
TEN	Very limited absorption, not detected in plasma	Hydroxylation	Not reported	Recovered unchanged in feces	[75,88]

## 6. *Alternaria* Mycotoxins Toxicity

The toxicological profile of *Alternaria* mycotoxins has been extensively studied using in vitro and, to a lesser extent, in vivo models. These compounds have been linked to a wide range of adverse effects, with outcomes strongly depending on the toxin type, concentration, exposure duration, and biological model employed.

Early investigations focused primarily on classical endpoints such as cytotoxicity and genotoxicity, which provide essential information on cell viability and DNA integrity, respectively. Research has been extended to mechanistic studies to reveal the underlying mode of action of mycotoxins (e.g., oxidative stress, topoisomerase inhibition, cell cycle arrest), as well as endocrine-related effects that highlight their potential to interfere with hormone signaling. Finally, because humans are typically exposed to mixtures rather than single compounds, co-exposure and interaction studies have gained increasing importance.

The following subsections summarize the current knowledge on these toxicological aspects on the liver and intestine, starting with cytotoxicity as the most fundamental endpoint.

### 6.1. Cytotoxicity

The cytotoxic effects of *Alternaria* mycotoxins have been investigated in a variety of intestinal and liver in vitro models, with results revealing a strong dependence on the specific compound, its concentration, the exposure duration, and the type of assay employed. Overall, *Alternaria* mycotoxins can be broadly divided into three categories based on the results: (i) highly cytotoxic compounds such as ATXs, (ii) moderately active compounds such as AOH and its monomethyl ether, and (iii) low- or non-cytotoxic compounds under the conditions tested, including TeA and TEN.

AOH and AME are among the most widely studied, particularly in intestinal cells. While short exposures (≤20 h) generally resulted in non-significant effects, longer incubations (≥24 h) consistently revealed reductions in cell viability in a concentration-dependent manner [89,90,91]. In Caco-2 cells, AOH induced up to 47% viability loss after 24–48 h exposure to ≥75 µM, with similar reductions observed at lower concentrations when exposure was extended to 72 h [92,93,94]. AME followed a similar trend, showing no effects in short-term assays, but clear viability decreases of ~30% at ≥25 µM after 24–48 h [94]. Interestingly, differences were noted across assays: Alamar Blue (AB)-based assays, which measure cellular redox activity, often failed to detect cytotoxicity at low concentrations, whereas MTT (mitochondrial dehydrogenase activity), SRB (sulforhodamine B, total protein content), and FC (flow cytometry) assays revealed more pronounced effects, suggesting that metabolic activity and protein synthesis may be more sensitive endpoints than redox-based readouts.

In hepatic models, AOH and AME displayed higher cytotoxic effect in HepG2 than in HepaRG cells, pointing to cell-line-dependent susceptibility. AOH showed moderate cytotoxicity with EC50 values of ~45 µM at 24 h with well-defined NOEL (3.9 µM) and LOEL (5.6 µM), while AME appeared more cytotoxic, reaching an EC50 of 18 µM at 24 h and with NOEL and LOEL values of 3.54 µM and 5.28 µM, respectively [95]. By contrast, HepaRG cells were more resistant, showing no clear effects even at 100 µM [96]. This difference may reflect variations in metabolic competence, since HepaRG cells retain more hepatocyte-like functions compared to the cancer-derived HepG2 line.

ATX-I and ATX-II stand out for their markedly higher cytotoxicity. Data on ATX-I are limited, but an EC50 value of 96 µM was reported in HepG2 cells [97]. In contrast, ATX-II consistently emerged as one of the most toxic *Alternaria* metabolites, with effects apparent at submicromolar levels. In HT29 colon cells, 24–72 h exposure caused strong viability loss with EC50 values as low as 0.8 µM [98,99]. In HepG2 cells, ATX-II also displayed high cytotoxicity, with an EC50 value of 7.3 µM at 24 h exposure [100]. Del Favero et al. (2018) showed that even 1 h exposure (0.1–10 µM) caused irreversible toxicity in human colonic HCEC cells; however, these effects were less pronounced in HT29, suggesting heightened susceptibility of non-transformed epithelia [101]. These results highlight ATX-II as a major contributor to cytotoxic risk.

ALT, TeA, and TEN present more variable outcomes. ALT showed strong cytotoxicity in HCT116 colon cells after 72 h exposure, with and IC50 of 3.13 µM [102]. By contrast, TeA was consistently less active, requiring high concentrations to elicit toxic effects. EC50 values exceeded 300 µM in both intestinal and hepatic cells after 24–48 h exposure [95], and while dose-dependent decreases were noted at these levels, the biological relevance of such high concentrations remains questionable. TEN, on the other hand, was repeatedly shown to be inactive across multiple cell lines and exposure times [91,96].

Taken together, these findings emphasize the importance of assay conditions (cell model, exposure time, endpoint measured) in evaluating cytotoxicity (Table 3).

Overall, current data suggest that ATX-II poses the highest cytotoxic concern due to its strong effects at submicromolar levels.

### 6.2. Genotoxicity

Genotoxic effects of *Alternaria* mycotoxins have been systematically assessed using several complementary approaches, including alkaline unwinding (AU), the comet assay (standard and Fpg-modified), and γ-H2AX detection. These assays collectively capture different levels of DNA damage, ranging from single- and double-strand breaks to oxidative base modifications and DNA repair signaling events.

#### 6.2.1. In Vitro Studies

For AOH, DNA strand breaks induction was observed at relatively short exposure times. Using the AU assay, significant DNA damage was detected in both intestinal (Caco-2, HT29) and hepatic (HepG2) cells within 1–1.5 h of exposure, with effects appearing at ≥ 6.25–12.5 µM, depending on the cell model [77,83]. Longer incubations (24 h) in HT29 cells failed to reproduce this effect, suggesting DNA repair activity [77]. In standard comet assays, DNA strand breaks were observed at 0.5–10 µM after 1 h exposure [98,99]. Moreover, the Fpg-modified comet assay, which additionally detects oxidative DNA lesions, did not detect an increase in DNA damage, indicating that oxidative base damage may not be the primary mechanism of AOH genotoxicity [98,99,103]. The γ-H2AX assay, which detects DNA double-strand breaks and activation of DNA repair pathways, showed phosphorylation of H2AX at ≥ 0.1 µM in HepG2 cells after 4 h exposure, but only in the absence of S9 metabolic activation, suggesting that AOH itself—and not its metabolites—acts as the primary genotoxic species [96].

AME showed generally similar, but slightly less genotoxic effects. AU assays detected DNA strand breaks in HT29 and HepG2 cells after 1 h exposure at ≥ 6.25 µM, though this effect was lost after 24 h in HT29, and required higher concentrations (≥25 µM) in HepG2 at the longer exposure [77]. Regarding comet assay results, one study reported DNA strand breaks at ≥ 10 µM (1 h exposure), while another observed no effects up to 50 µM after 3 h [98,104]. In the γ-H2AX assay, positive findings in HepG2 cells were observed at ≥ 1 µM after 4 h, but with more variability than AOH, and again with no clear evidence of oxidative DNA damage [96].

Altertoxins (ATX-I and ATX-II) appear more potent in inducing genotoxic effects. Both compounds caused DNA strand breaks in Caco-2 and HepG2 cells after 1–1.5 h of exposure, with ATX-I effects observed at 10 µM and ATX-II at 0.25 µM [83]. In HT29 cells, ATX-II induced DNA strand breaks in standard and Fpg-modified comet assays after 1 h at 0.05–0.1 µM [90,98,99], with positive results after 24 h exposure, suggesting more persistent DNA damage or slower repair [99]. ATX-I, in contrast, was evaluated only at 10 µM and exhibited lower genotoxicity than ATX-II, inducing a similar extent of DNA strand breaks to that caused by 1 µM ATX-II [83].

One other *Alternaria* mycotoxin, TeA, was negative in both comet and γ-H2AX assays, even at high concentrations (up to 200 µM), using short exposure times (1–4 h), regardless of metabolic activation [96,99].

Moreover, no experimental genotoxicity data could be identified for TEN or ALT, underscoring a significant gap in the toxicological characterization of these compounds.

Based on the available in vitro studies, ATX-II showed genotoxic effects at lower concentrations than the other *Alternaria* mycotoxins, whereas ATX-I, AOH, and AME produced DNA damage at somewhat higher concentrations [77,83,90,96,98,99,103,104]. The Fpg-modified comet assay results indicate that oxidative base damage contributes minimally to AOH and AME genotoxicity, while bulky adduct formation and replication stress may be more relevant, particularly for ATX-II. TeA did not induce detectable DNA damage under the studied conditions [96,99] (Table 4).

#### 6.2.2. In Vivo Studies

Although *Alternaria* mycotoxins have shown in vitro genotoxic effects in intestinal and hepatic cell models, these findings cannot be directly extrapolated to in vivo, as these in vitro models do not mimic the whole complexity of the in vivo system, and many times are based on tumor-derived cell lines—which have altered metabolism and DNA repair capacity. In vivo evidence confirming *Alternaria* mycotoxins genotoxicity remains limited.

In male NMRI mice, oral administration of AOH (to a limit of 2000 mg/kg bw) did not induce DNA damage in liver tissue, as assessed by the comet assay [72]. Similarly, male Sprague-Dawley rats exposed to AOH by gavage for 28 days (5.51–22.05 µg/kg bw/day) showed no significant DNA damage in liver tissue during treatment, although a transient increase in Pig-a mutant phenotype, an indicator of gene mutations in red blood cells, was observed after cessation of treatment, suggesting potential cumulative effects [105].

**Table 3:** Summary of in vitro cytotoxicity studies of *Alternaria* mycotoxins (AOH, AME, ATX-I, ATX-II, TEN, TeA) in liver and colon cell models. The table lists the assay used, exposure time, concentration ranges, cytotoxic effects, and corresponding references.

Mycotoxin	Assay	Cell Model		Time (h)	Concentration Range (μM)	Results	Ref.
AOH	AB	Colon	Caco-2	5, 20	0.01–40	No effect	[89,91,97]
			Caco-2	72	0–0.05	No effect	
		Liver	HepG2	72	0–0.05	No effect	
			HepG2	-	48.4–387.3	EC50 = 108.4 μM	
	FC	Colon	Caco-2	48	0.4–464.7	EC50 = 72.5 μM	[95]
		Liver	HepG2	24	0.4–464.7	EC50 = 45.64 ± 15.7 μM. NOEL = 3.9 μM. LOEL = 5.6 ± 0.2 μM	
	FDA	Colon	HCT116	24	10–200	IC50 = 65 μM. Hypoploid population goes from 9% (control) to 31%.	[106]
	LDH	Colon	HT29	1	0–50	No effect	[104]
	MTT	Colon	Caco-2	24	3.125–100	↓ Cell Viability ≥ 50 μM. Cytotoxicity reanged from 27–47%	[92,93,94,96,107,108]
			Caco-2	24, 48, 72	1.85–90	↓ Cell Viability ≥ 25 μM	
			Caco-2	24, 48, 72	12.5–100	↓ Cell Viability (in 40%) ≥ 75 μM after 24 and 48 h. At 72 h, ≥ 50 μM	
			Caco-2	24, 48	3.125–100	↓ Cell Viability (in 30%) ≥ 50 μM after 48 h.	
		Liver	HepG2	24	0.01–100	↓ Cell Viability at 100 μM	
			HepG2	24	3.2–72	↓ Cell Viability ≥ 12.8 μM	
			HepaRG	24	0.01–100	↓ Cell Viability at 100 μM	
	PC	Colon	Caco-2	24	3.125–100	↓ Protein content ≥ 25 μM ⟹ ↓ Cell Viability (in 25–32%)	[92,98,103]
			HT29	24	0.1–50	↓ Protein content at 50 μM (in 15%)	
			HT29	24	1–100	↓ Protein content ≥ 50 μM	
	WST-1	Colon	HT29	24	1–100	↓ Cell Viability ≥ 25 μM	[98,103]
			HT29	3	0.1–50	No effect	
AME	AB	Colon	Caco-2	72	0–0.05	No effect	[91,97]
		Liver	HepG2	72	0–0.05	No effect	
			HepG2	-	45.9–367.3	EC50 = 36 μM	
	FC	Colon	Caco-2	48	0.4–440.8	EC50 = 54.49 ± 30.54 μM	[95]
		Liver	HepG2	24	0.4–440.8	EC50 = 18 ± 1.84 μM NOEL = 3.54 μM LOEL = 5.28 ± 1.17 μM	
	FDA	Colon	HCEC-1CT	24	10–200	IC50 = 120 μM	[109]
	LDH	Colon	HT29	1	0–50	No effect.	[104]
	MTT	Colon	Caco-2	24, 48	3.125–100	↓ Cell Viability ≥ 25 μM. Maximum inhibition was 30%	[94,96]
		Liver	HepG2	24	0.01–100	↓ Cell Viability ≥ 10 μM. ~70% cytotoxicity at 100 μM	
			HepaRG	24	0.01–100	No effect	
ALT	SRB	Colon	HCT116	72	-	IC50 = 3.13 μM	[102]
ATX-I	AB	Liver	HepG2	-	35.5–283.8	EC50 = 96.5 μM	[97]
ATX-II	AB	Liver	HepG2	-	35.7–283.5	EC50 = 97.1 μM	[97]
	SRB	Colon	HT29	24	0.01–50	↓ Cell Viability ≥ 0.2 μM, dose-dependent	[98,99]
			HT29	24, 72	0.01–10	↓ Cell Viability ≥ 0.2 μM (24 h) and ≥ 0.01 μM (72 h). IC50 = 0.8 μM (72 h).	
	WST-1	Colon	HT29	24	0.1–25	EC50 = 16.5 μM	[100]
			HCEC-1CT	24	0.1–25	EC50 = 6.9 μM	
		Liver	HepG2	24	0.1–25	EC50 = 7.3 μM	
TeA	AB	Liver	HepG2	-	63.4–507.0	EC50 = 146 μM	[97]
	FC	Colon	Caco-2	48	0.5–608.4	EC50 = 356 ± 87.8 μM	[95]
		Liver	HepG2	24	0.5–608.4	EC50 = 146 ± 152.3 μM. NOEL = 15.57 ± 3.09 μM LOEL = 18.71 ± 0.56 μM	
	MTT	Liver	HepG2	24	0.01–100	↓ Cell Viability (60%) at 100 μM	[96]
			HepaRG	24	0.01–100	No effect	
TEN	AB	Colon	Caco-2	72	0–0.05	No effect	[91]
		Liver	HepG2	72	0–0.05	No effect	
	MTT	Liver	HepG2	24	0.01–100	No effect	[96]
			HepaRG	24	0.01–100	No effect	

AB (Alamar Blue)—measures metabolic activity through reduction of resazurin to fluorescent resorufin; FC (Flow cytometry)—quantifies cell death, viability, and apoptosis based on staining and scatter properties; FDA (Fluorescein Diacetate)—assess cell viability by esterase activity converting FDA into fluorescent fluorescein; LDH (Lactate dehydrogenase release)—detects membrane damage by measuring LDH leakage from dead cells; MTT—evaluates mitochondrial activity by conversion of MTT into insoluble formazan crystals; PC (Protein content)—estimates cell number or biomass by total protein quantification; SRB (Sulforhodamine B)—measures cellular protein content by dye binding to determine cell mass; WST-1—assess metabolic activity via cleavage of WST-1 tetrazolium salt to soluble formazan.

In male Sprague-Dawley rats, oral gavage of AME for 28 days (1.84–7.35 µg/kg bw/day) led to DNA damage at the higher doses tested (≥3.67 µg/kg bw/day), with partial recovery after a 14-day exposure-free period [110]. Additional endpoints indicated mutagenic and clastogenic activity in other tissues, although data for liver or GIT was limited [110].

ATX-I was administered to male Sprague-Dawley rats via oral gavage for 28 days (1.10–5.51 µg/kg bw/day) [111]. Histopathological changes were observed in the liver, including mild to moderate hepatocellular steatosis and cell swelling. Genotoxicity in liver tissue was evaluated by the comet assay and Pig-a mutation analysis, but no significant increases in DNA damage were observed, indicating that subchronic exposure at these doses does not induce direct hepatic genotoxicity [111].

Regarding ATX-II, this mycotoxin showed positive results in a study in male Sprague-Dawley rats using short exposure times and a single oral dose of 0.21 mg/kg bw. An increase in γH2AX levels was observed in colon and liver tissues, with more pronounced effect at 3 h and a decrease at 24 h post-exposure, suggesting transient DNA double-strand breaks [112].

Overall, in vivo data indicates that AOH and ATX-I show low or no genotoxicity in liver and colon under the tested conditions, while AME and ATX-II induce DNA damage. The coexistence of hepatic histopathological changes without DNA strand breaks in ATX-I exposure and, conversely, a transient DNA damage response in ATX-II exposure, underscore the need for mechanistic studies to elucidate these mycotoxins’ modes of action.

**Table 4:** Summary of in vitro genotoxicity studies of *Alternaria* mycotoxins (AOH, AME, ATX-I, ATX-II, TEN, TeA) in liver and colon cell models. The table lists the assay used, exposure time, concentration ranges, DNA damage effects, and corresponding references.

Mycotoxin	Assay	Cell Model		Time (h)	Concentration Range (uM)	Results	Ref.
AOH	Alkaline unwinding	Colon	Caco-2	1.5	10	Positive	[77,83]
			HT29	1	1–25	Positive ≥ 6.25 μM	
			HT29	24	5–25	Negative	
		Liver	HepG2	1	12.5–50	Positive ≥ 12.5 μM	
			HepG2	1.5	10	Positive	
	Comet	Colon	Caco-2	24	15–60	Positive ≥ 15 μM.	[90,98,99,103,104,113]
			HT29	1	0.1–50	Positive ≥ 10 μM.; no difference ± Fpg	
			HT29	1	0.1–50	Positive ≥ 0.5 μM; no difference ± Fpg	
			HT29	1	50	Positive	
			HT29	3	50	Negative	
	γH2AX	Liver	HepG2	4	0.1–100	Positive without S9, negative with S9.	[96]
AME	Alkaline unwinding	Colon	HT29	1	1–25	Positive ≥ 6.25 μM	[77]
			HT29	24	5–25	Negative	
		Liver	HepG2	1	6.25–50	Positive ≥ 6.25 μM	
			HepG2	24	5–25	Positive ≥ 25 μM	
	Comet	Colon	HT29	1	1–50	Positive ≥ 10 μM.; no difference ± Fpg	[98,104]
			HT29	3	0.1–50	Negative	
	γH2AX	Liver	HepG2	4	0.1–100	Positive without S9	[96]
ATX-I	Alkaline unwinding	Colon	Caco-2	1.5	10	Positive	[83]
		Liver	HepG2	1.5	10	Positive	
ATX-II	Alkaline unwinding	Colon	Caco-2	1.5	0.25–1	Positive ≥ 0.25 μM	[83]
		Liver	HepG2	1.5	0.25–1	Positive ≥ 0.25 μM	
	Comet	Colon	HT29	1	1	Positive	[90,99,103]
			HT29	1	0.01–1	Positive ≥ 0.1 μM; earlier response with Fpg (≥0.05 μM)	
			HT29	1	0.01–1	Positive ≥ 0.1 μM; earlier response with Fpg (≥0.05 μM)	
			HT29	24	0.01–1	Positive ≥ 0.1 μM; earlier response with Fpg (≥0.05 μM)	
TeA	Comet	Colon	HT29	1	0.2–200	Negative	[99]
	γH2AX	Liver	HepG2	4	0.1–100	Negative with and without S9	[96]

AU (Alkaline unwinding) assay—detects single-strand breaks; Comet assay—measures DNA strand breaks, with the enzyme Fpg it also detects oxidative DNA damage by converting oxidized purines into detectable strand breaks; γH2AX assay—identifies DNA double-strand breaks via histone H2AX phosphorylation at damage sites.

### 6.3. Mechanistic Insights in Intestinal and Liver Models

In addition to the cytotoxic and genotoxic effects described above, some research has been performed aiming to undestand the mechanistic mode of action of these mycotoxins.

In intestinal models, both AOH and AME have been shown to interfere with epithelial homeostasis by disturbing cell cycle progression and inducing programmed cell death. In Caco-2 and HT29 cells, exposure led to G2/M arrest, reduced proliferation, and increased apoptosis/necrosis, indicating that their prolonged presence in the intestinal lumen may compromise epithelial renewal capacity [73,106,107,109,114,115].

**Figure 3:**
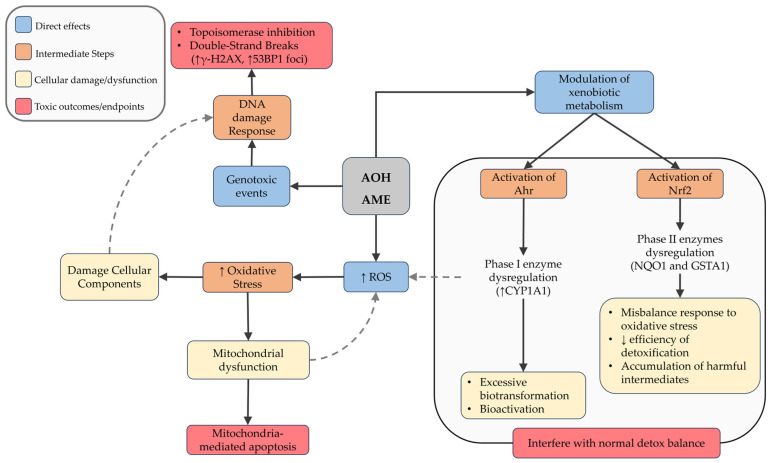
Schematic representation of the main cellular mechanisms induced by *Alternaria* mycotoxins AOH and AME. Upon exposure, these compounds can increase reactive oxygen species (ROS) and modulate xenobiotic metabolism via activation of Ahr and Nrf2 pathways. These events contribute to oxidative stress, DNA damage, mitochondrial dysfunction, and ultimately lead to apoptosis or disruption of the cellular detoxification balance. Dashed arrows indicate feedback or secondary amplifying interactions between pathways.

In hepatic models, both toxins induced DNA strand breaks, phosphorylation of H2AX, and activalion of cell cycle checkpoints [24,42,46,57]. These effects support topoisomerase I/II poisoning as a central mechanism, consistent with replication-associated DNA double strand breaks [96,104,116,117,118]. Beyond DNA damage, AOH and AME may modulate redox balance and xenobiotic metabolism, by activation of the aryl hydrocarbon receptor (AhR) that leads to overexpression of phase I enzyme CYP1A1, potentially generating reactive intermediates [119]. Moreover, both AOH and AME seem to modulate the Nrf2 pathway, altering the expression of antioxidant and phase II detoxification enzymes, including NAD(P)H:quinone oxidoreductase 1 (NQO1) and glutathione S-transferase alpha 1 (GSTA1) [120,121,122]. However, the absence of oxidative base lesions in Fpg-modified comet assays [59,60] suggests that ROS are not the primary driver of neither AOH nor AME genotoxicity but may act as a secondary stress pathway that amplifies DNA damage (Figure 3).

**Figure 4:**
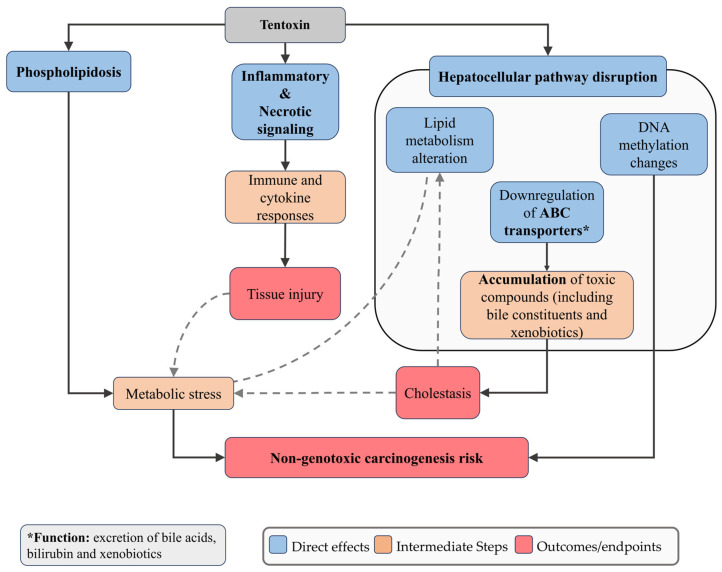
Schematic representation of the main cellular mechanisms induced by Tentoxin. TEN downregulates ATP-binding cassette (ABC) transporters, impairing efflux of bile acids, bilirubin, and xenobiotics, which compromises hepatocellular detoxification and predisposes cells to cholestasis, accumulation of toxic bile constituents, and apoptosis. TEN also modulates genes associated with inflammatory and necrotic pathways, contributing to hepatocyte injury despite limited genotoxic effects. Dashed arrows indicate feedback or secondary amplifying interactions between pathways.

TEN, in contrast, has exhibited relatively low cytotoxicity and genotoxicity, but demonstrated pronounced hepatotoxicity. In HepaRG cells, TEN caused a concentration-dependent downregulation of ATP-binding cassette (ABC) transporters, which are essential for the efflux of bile acids, bilirubin, and xenobiotics [96]. When these transporters are reduced, bile components cannot be efficiently removed from hepatocytes and instead accumulate inside the cells. This buildup interferes with normal bile flow (cholestasis) and places stress on hepatocytes, which can lead to inflammation and cell injury. Furthermore, TEN altered the expression of genes associated with inflammatory and necrotic pathways, supporting its role in hepatocyte injury despite limited evidence of genotoxicity [96,123] (Figure 4).

**Figure 5:**
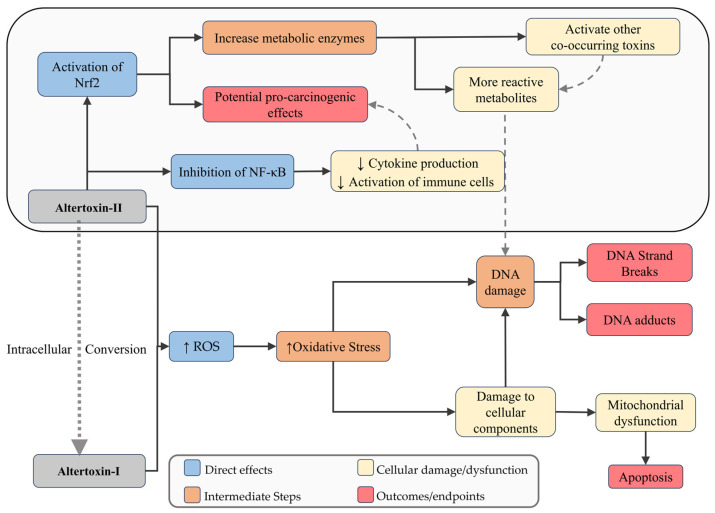
Schematic representation of the main cellular mechanisms induced by Altertoxins. These compounds induce oxidative stress, mitochondrial dysfunction, and apoptosis. They form bulky covalent DNA adducts causing replication and transcription interference. ATX-II additionally acts as topoisomerase II poison, stabilizing the cleavage complex and inducing double-strand breaks, and activates the Nrf2-ARE pathway, leading to antioxidant gene induction. Dashed arrows indicate feedback or secondary amplifying interactions between pathways.

Altertoxins (ATX-I and ATX-II) have been shown to induce oxidative stress, mitochondrial dysfunction, and apoptosis in HT29 and HepG2 cells [90,98,119]. ATXs also formed bulky covalent DNA adducts that interfere with replication and transcription, which were associated with γ-H2AX accumulation in intestinal cells (HCEC, HT29) [101,104,124]. Mechanistically, ATX-II functions as a topoisomerase II poison by stabilizing the cleavage complex and inducing DSBs in HT29 cells, a feature it shares with AOH [96,104]. Interestingly, ATX-II—but not ATX-I—activates the Nrf2-ARE pathway, as evidenced by increased Nrf2 nuclear accumulation and induction of downstream genes, such as γ-glutamate cysteine ligase, the rate-limiting enzyme in glutathione synthesis [73]. This dual role in both depleting and subsequently inducing antioxidant defenses highlights the complex redox-related toxicity of ATX-II (Figure 5).

A comparative overview of the toxicological characteristics of key *Alternaria* mycotoxins is presented in Table 5.

Although several studies exist on the toxicity of *Alternaria* toxins, the toxicological properties of the metabolites of these toxins themselves still represent a significant research gap. Through advanced in silico tools, including metabolic prediction algorithms and quantitative structure–activity relationship (QSAR) models, aiming to characterize the potential biotransformation pathways and toxicodynamics of AOH, twelve likely metabolites resulting from both phase I (oxidative, reductive, and hydrolytic) and phase II (conjugative) reactions were identified [125]. Predicted phase I metabolites included hydroxylated and methylated derivatives, while phase II metabolism favored glucuronidation and sulfation, consistent with detoxification pathways observed for other phenolic xenobiotics. Toxicity modeling suggested that several of these metabolites—particularly certain hydroxylated and methylated derivatives—may retain or even potentiate the toxicological properties of the parent compound. Specifically, endpoints such as cytotoxicity, genotoxicity, endocrine-disruptive activity, nephrotoxicity, and vascular toxicity were predicted to persist in some metabolites, indicating incomplete detoxification. The study underscored that biotransformation of AOH could generate both detoxified conjugates and reactive intermediates capable of interacting with DNA or hormonal receptors, thus complicating the toxicokinetic profile of the compound. From a risk assessment standpoint, the authors concluded that these findings highlight a crucial data gap: the absence of empirical toxicological data on AOH metabolites. They advocate for targeted in vitro and in vivo studies to validate the predicted toxicity and to determine whether metabolic conversion mitigates or exacerbates the overall toxic burden of *Alternaria* contamination [125].

## 7. Co-Exposures, Mixtures, and Real-World Relevance

Given that multiple mycotoxins often co-occur in contaminated food, it is essential to consider not only their individual toxicities, but also their combined effects. As Crudo et al. (2019) emphasize, *Alternaria* mycotoxin contamination should be evaluated within the broader context of combined mycotoxin exposure rather than in isolation [25]. While literature on whether these compounds interact in ways that increase overall toxicity remains limited, available studies suggest that combined exposure can lead to additive or even synergistic cytotoxic effects, particularly at low doses.

For instance, AOH displayed enhanced toxicity in the presence of some *Fusarium* mycotoxins (3- and 15-acetyldeoxynivalenol (ADON)) in Caco-2 cells, suggesting that its effects may be potentiated in co-exposures [108]. Additionally, AOH and AME have shown additive cytotoxicity on HCT116 cells when combined in a 1:1 ratio [126].

The interaction between AOH and ATX-II is particularly interesting. In liver (HepG2) and colon (HT29, HCEC-1CT) cells, ATX-II generally displayed higher cytotoxicity than AOH [100]. When applied in combination, a 1:1 mixture (750 nM each) produced greater cytotoxicity in HepG2 cells than the calculated expectation, indicating a synergistic effect. In contrast, antagonistic effects were observed at 1:10 ATX-II:AOH ratios, with reduced cytotoxicity compared to predicted additive outcomes. These antagonistic interactions were detected at 5 µM:50 µM in HepG2 and HCEC-1CT cells, and at 10 µM:100 µM in HT29 cells, with the strongest effects in HepG2 cells [62]. Such dose-dependent and cell-type-specific interactions highlight the complexity of *Alternaria* mycotoxin mixtures. While antagonism at high concentrations is unlikely to be relevant for typical dietary exposure, additive or synergistic effects at low levels are more plausible and toxicologically significant.

In vivo data on the combined effects of *Alternaria* mycotoxins is very limited, yet available findings indicate that oral exposure to mixtures, such as ALT, AOH and TeA can lead to cumulative hepatic effects. Following prolonged exposure, no evident structural alterations in the gastrointestinal tissues of rats have been reported, suggesting that the mixture can pass through the intestinal tract without causing local damage under the tested conditions. However, once absorbed, increases in serum indicators of liver injury and corresponding morphological changes in hepatic tissue have been observed, demonstrating that the liver is a primary target organ during sustained intake of these toxin combinations [127]. Moreover, exposure to complex mixtures of *Alternaria* toxins has enhanced changes in liver metabolism, with the accumulation of short-chain acylcarnitines and downregulation of riboflavin, which may impair β-oxidation and coenzyme-dependent metabolic pathways, as compared with isolated ATX-II [71].

A summary of the in vitro combined toxicity effects of key *Alternaria* mycotoxins is presented in Table 6.

## 8. Conclusions

The occurrence of *Alternaria* mycotoxins in food and feed is highly variable, shaped by climate, agricultural practices, storage, and processing conditions. Major sources include tomato products, cereals, sunflower seeds, and processed foods. Among these toxins, TeA frequently appears at the highest concentrations in monitoring surveys, whereas AOH and AME are of particular concern because estimated dietary intakes—especially in toddlers—can exceed the TTC. Co-occurrence within single samples is frequent, raising the likelihood of additive or synergistic effects. Contaminated feed further extends exposure pathways, linking animal and human risk.

From a toxicological perspective, the GIT and liver include the target organs first exposed after ingestion. The limited absorption of the parent compounds of several mycotoxins suggests that epithelial and hepatic cells may experience direct cytotoxic and genotoxic effects, potentially compromising epithelial integrity and metabolic function. For absorbed fractions, biotransformation through Phase I/II pathways may both detoxify or generate reactive intermediates, influencing systemic toxicity. These organ-specific susceptibilities make the colon and liver central to understanding toxicokinetic and toxicodynamic outcomes.

Mechanistically, some *Alternaria* toxins, such as AOH and AME, seem to exert genotoxicity primarily via topoisomerase poisoning, whereas others, like ATXs, may act through distinct pathways that involve oxidative stress and DNA damage, independent of topoisomerase inhibition. Xenobiotic metabolism is also affected, with modulation of Phase I and II detoxification systems, reflecting dual disruption of redox balance and detoxification capacity. This diversity of modes of action highlights the potential for additive or synergistic effects under co-exposure scenarios.

Taken together, these findings highlight the predominance of data gathered from in vitro studies using cancer-derived cells. Thus, there is the need for other systematic studies that employ non-cancer-derived and non-transformed cell lines, as primary cultures or induced pluripotent stem cells (iPSCs). Also, more complex biological systems are needed, as co-cultures, organoids, and in vivo models, exposed to physiologically relevant mycotoxin concentrations, with attention to both parent toxins and metabolites. Given their widespread occurrence and potential health implications, integrated risk assessment strategies that combine occurrence data with toxicokinetic and mechanistic insights remain essential to guide regulatory action and mitigation strategies, aimed at reducing human exposure.

## Figures and Tables

**Table 5:** Overview of the in vitro cytotoxicity, genotoxicity, molecular mechanisms, and key observations reported for major *Alternaria* mycotoxins.

Mycotoxin	Cyt.	Gen.	Mechanisms	Notes	Ref
ALT	Moderate	Low	Possibly oxidative stress	Limited data, but current evidence suggests low toxicity	[79,87]
AOH	Moderate to High	Moderate	DNA strand breaks, inhibition of topoisomerase II, oxidative stress, activation of p53, AhR → ↑ CYP1A1, modulation of Nrf2 pathway	One of the best-studied; shows both direct and indirect genotoxic effects	[73,90,96,98,104,114,115,116,119,120,121]
AME	Moderate to High	Moderate	Similar to AOH: topoisomerase II inhibition. γ-H2AX/53BP1 foci, AhR activation, oxidative stress, Nrf2 and phase II enzyme modulation	Often co-occurs with AOH and mimics many of its toxic mechanisms	[73,77,78,80,90,96,104,119,120,121,122]
ATX-I	High	High	DNA adduct formation, oxidative stress, apoptosis, γ-H2AX ↑	Higher DNA damage than AOH;	[90,98,101,104,119,124]
ATX-II	Very High	Very High	Inhibits topoisomerase II, causes double-strand breaks, DNA adducts, oxidative stress, rapid metabolism to ATX-I	The most genotoxic *Alternaria* toxin; acts mainly in the gut	[90,98,101,104,119,124]
TeA	High	Low	Inhibits protein synthesis by targeting eukaryotic initiation factors, induces apoptosis, oxidative stress	Not genotoxic, but very cytotoxic	[96,99]
TEN	Low	Low to negligible	Downregulation of ABC transporters in hepatic cells, which leads to impaired bile and xenobiotic excretion, affects inflammatory and apoptotic gene expression	Mainly hepatotoxic; indirect mechanisms	[96,119,123]

Cyt—Cytotoxicity; Gen—Genotoxicity; AhR—Aryl hydrocarbon receptor; CYP1A1—Cytochrome P450 1A1; Nrf2—Nuclear factor erythroid 2–related factor 2; γ-H2AX—phosphorylated H2AX histone variant; 53BP1—p53-binding protein 1.

**Table 6:** Summary of the in vitro cytotoxicity and interaction type observed for different combinations of *Alternaria* mycotoxins.

Toxin Combination	Cell Line	Observed Combined Mechanistic Effect	Interaction Type	Ref.
AOH + AME (1:1)	Caco-2	**↑** cytotoxicity compared to single exposure. AOH + AME induced oxidative stress, apoptosis. Quercetin attenuated ROS and cytotoxicity, suggesting oxidative stress is a key mechanism.	Synergistic	[94]
	HCT116	**↑** cytotoxicity. DNA damage: increased micronuclei formation and genotoxic stress. Oxidative stress: ↑ ROS.	Additive	[126]
AOH + *Fusarium* toxins (3- and 15-ADON)	HepG2	**↑** cytotoxicity than AOH alone. Mechanistic endpoints: apoptosis induction, mitochondrial membrane potential disruption, partial formation of degradation products affecting toxicity.	Low-dose binary combinations showed additive effects; higher concentrations showed antagonism.	[108]
AOH + ATX-II (1:1)	HepG2, HT29, HCEC-1CT	**↑** cytotoxicity at higher concentrations. Mechanistic effects: modulation of miRNA expression (e.g., miR-34a), apoptosis induction, DNA damage.		[100]

ADON—acetylated derivatives of deoxynivalenol.

## Data Availability

No new data were created or analyzed in this study.

## Abbreviations

The following abbreviations are used in this manuscript:

ALT	Altenuene
AME	Alternariol monomethyl ether
AOH	Alternariol
AT	*Alternaria* Toxin
ATX	Altertoxin
TeA	Tenuazonic acid
TEN	Tentoxin
AU	Alkaline Unwinding
SRB	Sulforhodamine B
FC	Flow cytometry
PC	Protein Content
GIT	Gastrointestinal tract
